# Sleep Stage Estimation from Bed Leg Ballistocardiogram Sensors

**DOI:** 10.3390/s20195688

**Published:** 2020-10-05

**Authors:** Yasue Mitsukura, Brian Sumali, Masaki Nagura, Koichi Fukunaga, Masato Yasui

**Affiliations:** 1 Department of System Design Engineering, Faculty of Science and Technology, Keio University, Yokohama 223-8522, Japan; brian.sumali@keio.jp (B.S.); nagura@mitsu.sd.keio.ac.jp (M.N.); 2 Department of Internal Medicine, School of Medicine, Keio University, Tokyo 160-8582, Japan; k-fuku@jf7.so-net.ne.jp; 3 Department of Pharmacology, School of Medicine, Keio University, Tokyo 160-8582, Japan; myasui@a3.keio.jp

**Keywords:** biomedical informatics, medical information systems, biomedical equipment, biomedical signal processing, cardiography

## Abstract

Ballistocardiogram (BCG) is a graphical representation of the subtle oscillations in body movements caused by cardiovascular activity. Although BCGs cause less burden to the user, electrocardiograms (ECGs) are still commonly used in the clinical scene due to BCG sensors’ noise sensitivity. In this paper, a robust method for sleep time BCG measurement and a mathematical model for predicting sleep stages using BCG are described. The novel BCG measurement algorithm can be described in three steps: preprocessing, creation of heartbeat signal template, and template matching for heart rate variability detection. The effectiveness of this algorithm was validated with 99 datasets from 36 subjects, with photoplethysmography (PPG) to compute ground truth heart rate variability (HRV). On average, 86.9% of the inter-beat intervals were detected and the mean error was 8.5ms. This shows that our method successfully extracted beat-to-beat intervals from BCG during sleep, making its usability comparable to those of clinical ECGs. Consequently, compared to other conventional BCG systems, even more accurate sleep heart rate monitoring with a smaller burden to the patient is available. Moreover, the accuracy of the sleep stages mathematical model, validated with 100 datasets from 25 subjects, is 80%, which is higher than conventional five-stage sleep classification algorithms (max: 69%). Although, in this paper, we applied the mathematical model to heart rate interval features from BCG, theoretically, this sleep stage prediction algorithm can also be applied to ECG-extracted heart rate intervals.

## 1. Introduction

When the heart beats, blood inside the aortic vessels are pushed, causing expansions and contractions in response to the blood flow. This causes feeble, rhythmical involuntary movements in the body that can be detected by Ballistocardiograms (BCGs) [1]. The possibility of noninvasive and nonintrusive measurement is a big advantage of BCG systems as conventional techniques for measuring cardiac activities, which commonly require the subject’s body to be temporarily attached to electrodes, textiles, or other sensors. BCG research took off in 1877, when the performance of the circulatory system was shown to be interpretable from BCG signals. Despite this, there were two main problems in early BCG studies. The first problem was the mechanical limitation of the measurement devices. The accuracy of early BCG sensors was poor, resulting in high measurement errors. The second problem was the non-uniformity of the units. Uniformity of the units are especially important in the field of data science and normalization is needed if the obtained data have different ranges. Studies in those days measured vibration using different devices and different nomenclatures. The American Heart Association’s Committee on Ballistocardiographic Terminology resolved these problems by defining the standards for spatial axes and conventions for polarity.

Over time, BCG devices with high accuracy and fast computation were developed and, with the two main problems in BCG field resolved, the number of studies increase and BCGs have recently been gaining significance in cardiological examinations [1,2]. Of late, studies have also attempted to utilize BCGs for home medicine or e-health because of the need for privacy, unobtrusiveness, and ubiquitous computing [1,2,3,4,5,6]. In those studies, various sensors were utilized, such as chair [1], weight scale [6], and sheet-type sensors [2,3,4,5,7]. The sheet-type sensor is commonly placed on a bed and is especially useful for continuous heart rate monitoring during sleep [1]. Moreover, a lot of unobtrusive and continuous ECG measuring methods with wearable devices have been proposed in the field of Body Sensor Networks (BSNs) in recent years [8,9,10,11,12,13,14]. In association with them, many kinds of signal processing methods and a framework based on wearable sensors and energy-efficient communication algorithms are proposed in the field [14,15,16,17,18,19,20,21,22,23,24,25,26,27].

As opposed to these BSN studies, our system’s novelty lies in the fact that it uses a signal called BCG that can be measured without wearing any sensors. However, BCG tends to contain noise, and uncertainty exists in the measurement signals, so an advanced signal processing method is indispensable. Compared with the conventional sheet-type BCG system, our proposed system aims to enable accurate measurement regardless of posture and the patient’s position when lying down on a bed by using the four sensors in combination. This paper proposes the prominence heart rate variability measurement system and algorithm.

Conventional studies indicated that heart rate variability (HRV) techniques are useful for monitoring sleep stage. Versace et al. utilized electrocardiograms (ECGs) to analyze heart rate variability (HRV) in sleeping subjects. The low-frequency HRV power and heart rates were relatively lower during deep sleep compared to rapid eye movement (REM) sleep [24]. After their success, several studies proposed sleep stage estimation algorithms from ECG-extracted HRVs [25,26]. These studies suggested the possibility for sleep stage estimation using BCGs, as HRV can also be extracted from BCGs. By performing an all-night continuous BCG measurement, sleep stage estimation using BCG was made possible [2,7].

Conventional studies of sleep stage classification tend to have low accuracy for in this respect, especially for five-stage classification. In recent years, Wei et al. [28] and Radha et al. [29] proposed a deep learning approach for sleep stage classification, utilizing heart rate features. The authors of [30] compare different machine learning models utilizing ECG features. Studies tend to group some sleep stages together to reduce the problems, yet still fail to produce accurate classifications. Therefore, we aimed to construct mathematical models for detecting a subject’s sleep stage using only the BCG signals as an additional objective.

Although BCG sensors mounted in bed legs are said to be the most nonintrusive BCG sensors, some challenges must be solved before they can be implemented in real sleep studies. The most challenging problem when utilizing sensors mounted in bed legs is the noise caused by subject’s arbitrary sleeping posture. Good and reliable HRV extraction algorithm is essential for solving this problem. The objectives of this study are twofold: to develop a heartbeat detection algorithm based on BCG signals from bed sensors for the purpose of daily monitoring and to obtain a mathematical function to predict a person’s sleep stage from their heartbeat.

The rest of this paper is structured as follows: in Section 2, we present a novel bed sensor system and an evaluation metric for the corresponding system. Details of the heartbeat detection algorithm and the mathematical model for sleep stage prediction are reported in Section 3. In Section 4, the novel algorithm is evaluated, and the results are described. The mathematical model for sleep prediction is also explained in this section. The results are discussed in Section 5 and the paper is concluded in Section 6.

## 2. Materials and Methods

### 2.1. Subjects

Two datasets were prepared for this study. The recruitment for the datasets was performed separately. The only criterion for the participants was to be of healthy state, both physically and mentally, certified by a physician. For the first step of HRV extraction, this study recruited 39 healthy subjects (male: 36, female: 3) as the test subjects for validating the bed sensor system. Three of the subjects’ data were excluded (subject 11, 18 and 22) as they were found to be afflicted with sleep apnea. Ten minutes of continuous BCG recording of each subject was performed in each of three postures (lying down on the back, left, and right side). Pulse oximetry was used during the measurement for recording photoplethysmography (PPG) data. It was attached to the subject’s fingertip to serve as the baseline. The details of the data used in the analysis are described in Table 1. Some of the recordings did not have the PPG data and were not used for analysis in this study. The second dataset was utilized for the second step of sleep stage prediction. Twenty-five healthy subjects (male: 19, female: 6) were recruited. Polysomnography (PSG) signals of electroencephalograpm (EEG), electro-oculogram (EOG), electromyogram (EMG), ECG, respiratory flow, and pulse oximetry (SpO2) were obtained. The subjects were instructed to sleep with a natural sleeping posture. Trained experts labeled the data with sleep stages to serve as the baseline for sleep stage prediction.

### 2.2. Bed Sensor Architecture

The sensor attached to the bed used in this study consisted of a dedicated sensor unit, a data logger and an on-pre-server (hereinafter referred to as an on-server). The device detected the user’s activity on the bed and the subtle changes in the load were captured as electric signals. This electric signal is converted into useful information by the digital processing by performing Analog-to-Digital (AD) conversion with the data logger. The information obtained by the data logger is then transferred to the on-server via the wireless Local Area Network (LAN). The aforementioned information is accessible from the display monitor connected to the on-server. It can also be viewed from the PC or mobile terminal via an online browser.

The technical specifications of dedicated sensor units that can be installed under bed legs are as follows:

1. Load characteristics:
  Rated capacity (R.C.): 100 kg
  Allowable overload: 150% R.C.
  Marginal overload: 200% R.C.
  Rated output (RO): 2 mV/V ± 0.2 mV/V Nonlinearity: 0.030% R.O.
  Hysteresis: 0.040% R.O.
  Repeatability: 0.020% R.O.
  Creep: 0.028% RO/20 min
2. Electrical characteristics:
  Recommended applied voltage: 12 V or less
  Maximum applied voltage: 20 V
  Zero balance: ±0.10 mV/V
  Input resistance: 185 Ω to 235 Ω
  Output resistance: 170 Ω to 180 Ω
  Insulation resistance: 2000 M Ω or more (DC 50 V)
3. Temperature characteristics:
  Temperature compensation range: −10 °C to 40 °C
  Allowable temperature range: −10 °C to 40 °C
  Temperature effect of zero: 0.056% RO/10 °C
  Temperature effect of output: 0.017% LOAD/10 °C
4. Other:
  Material of flexure body: aluminum alloy

**Figure 1:**
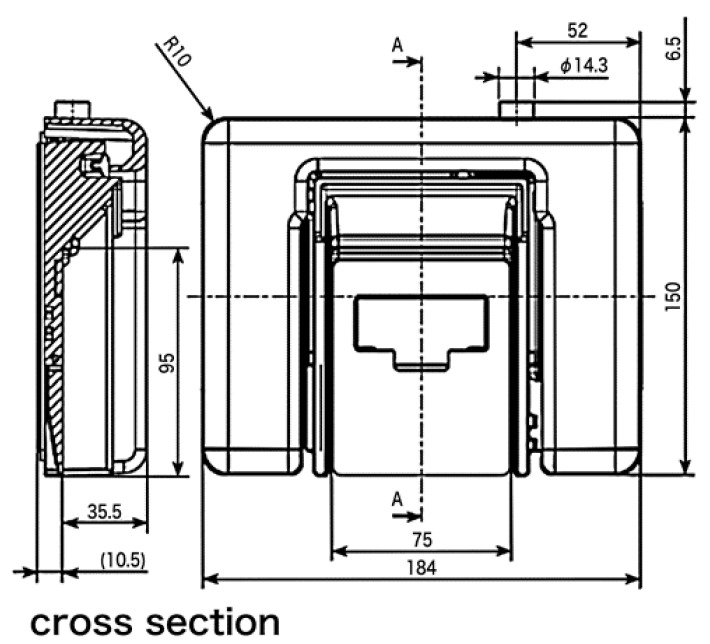
Sensor shape used in this paper. Figure courtesy of MinebeaMitsumi Inc.

Figure 1 shows the details of the sensor shape.

### 2.3. Bed Sensors

**Figure 2:**
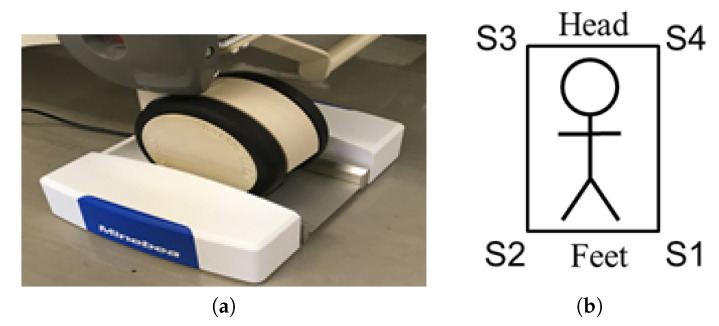
Bed sensor system: (**a**) a photograph of the bed leg sensor used in this study; this sensor was placed under each of the bed leg. Figure courtesy of MiuebeaMitsumi Inc; (**b**) the ID numbers assigned to each sensor.

In this study, bed-leg sensors were utilized for BCG measurement. These sensors were placed under four legs of an ordinary bed to detect subtle vibrations from subjects lying on the bed. The sensors are depicted in Figure 2a. As the device is compact in size, it does not cause inconvenience to users during daily life. These sensors have the same characteristics and can detect vibrations as fluctuations in voltage with a sampling rate of 200 Hz. Figure 2b shows the bed sensor system and ID given to the sensors.

This system enables BCG measurement as long as the subjects are lying on a bed. In addition, any bed can be used in conjunction with these sensors. These advantages show the usefulness of this system.

**Figure 3:**
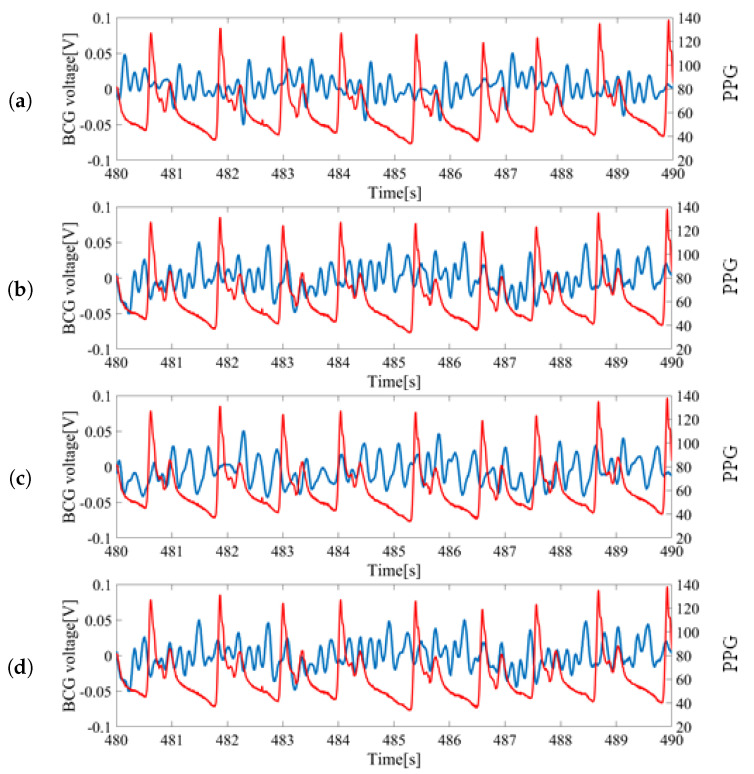
Sample data of raw sensor inputs measured from a subject lying on the back: (**a**) Ballistocardiogram (BCG) signal from S1 vs. photoplethysmography (PPG) signal; (**b**) BCG signal from S2 vs. PPG signal; (**c**) BCG signal from S3 vs. PPG signal; (**d**) BCG signal from S4 vs. PPG signal. Blue colored signals correspond with the measurement from bed leg sensors and red colored signals (finger pressure) correspond to the PPG signal from the pulse oximeter.

The raw inputs of BCG sensors and the pulse oximeter from a subject lying on their back are shown in Figure 3. The measurement was simultaneous and synchronized between the BCGs and PPG. The PPG signal was used as the baseline for timing the heartbeat and computing the HRV.

For each wave, the largest peak was assumed to be the heartbeat and was utilized for HRV estimation. The inputs from S1 and S2 located at the feet side were ideal as the peaks could be clearly identified. However, the inputs from S3 and S4 located at head side had low signal-to-noise ratios (SNR) and the peaks were difficult to locate. Thus, signals from S1 and S2 sensors were mainly used for the analysis.

## 3. Analysis

**Figure 4:**
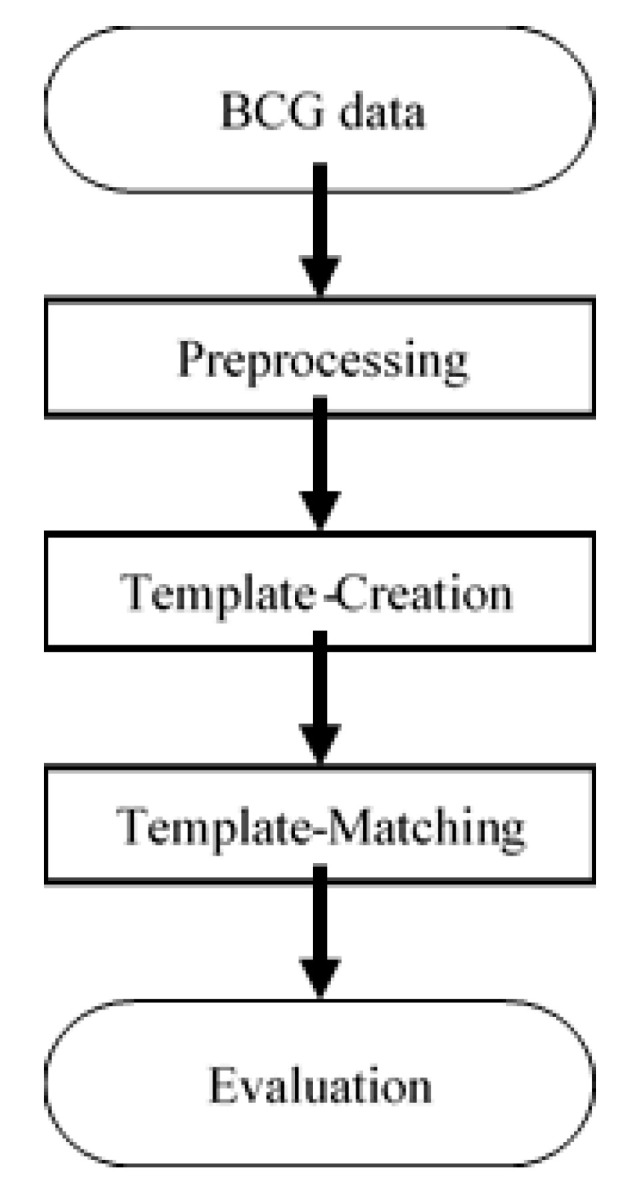
Flowchart of the proposed HRV detection algorithm.

In this section, the data analysis method is described. A template-matching algorithm was utilized to find the largest peaks. This approach mainly consisted of three steps: preprocessing, template creation, and template matching. The flow chart of our heartbeat detection algorithm is shown in Figure 4 and the details and evaluation method are described below. The mathematical equations for predicting sleep stages are described afterwards.

### 3.1. Preprocessing

In this step, two types of preprocessing were applied to the raw BCG data. First, a Butterworth bandpass filter with a cutoff frequency at 1 Hz and 8.5 Hz was utilized to remove low-frequency noise caused by respiration. Afterwards, the normalization was performed, such that the value ranges from −0.5 to 0.5. The dataset is then split into two sets: a training set and an evaluation set. The training set consists of 49 datasets from 18 subjects (subject #1 to #20). The holdout set consists of 50 data from 18 subjects (subject #21 to #39). This validation was performed to simulate real-world cases where no information on new subjects is known.

### 3.2. Template Creation

**Figure 5:**
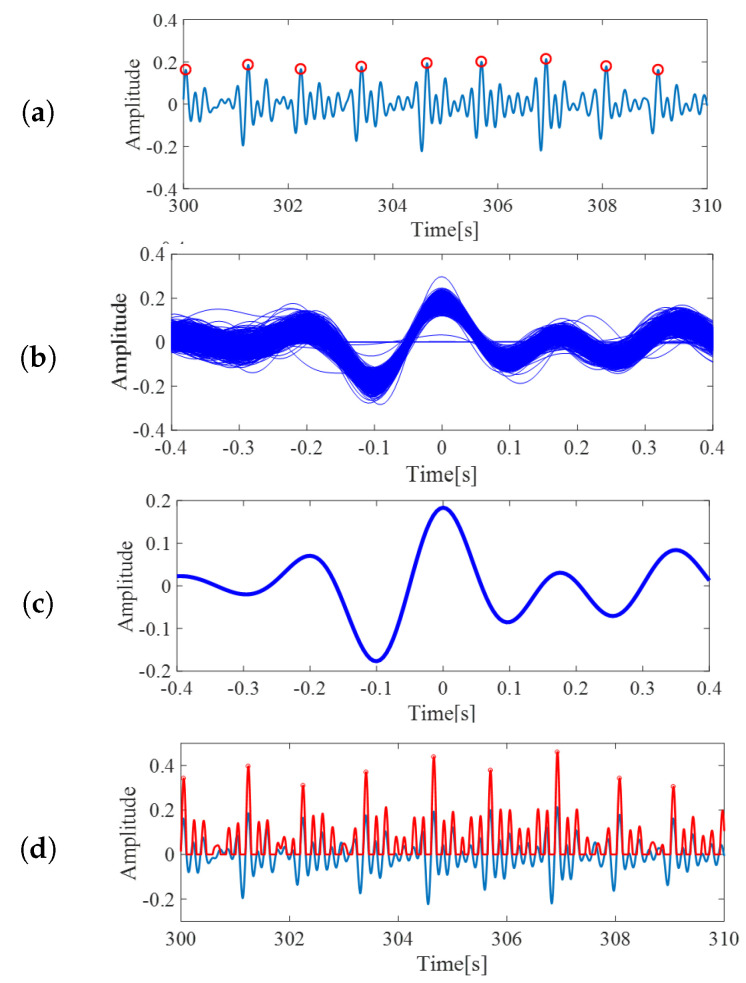
(**a**) The red circles are local maxima from BCG inputs, from the template creation step. (**b**) The extracted segments, which were assumed to represent BCG wave form. (**c**) BCG template created by averaging the extracted segments. (**d**) The correlation coefficients of pre-processed BCG and the template. Value of correlation becomes high around BCG peaks. These peaks are defined as heartbeats.

A single template was prepared from the training set using the following process. First, local peaks with at least 0.7 s intervals were extracted from the signal, as shown in Figure 5a. The minimum peak distance was 0.7 s and was chosen by means of empirical observation. We randomly selected 20 out of 39 subjects as the training group, and the remaining 19 subjects as the evaluation group. Then, we tested the minimum distances of the peaks as parameters, from 0 s to 1 s, with 0.1 s steps, and concluded that 0.7 s was the most suitable.

Next, for each peak, the data from 0.4 s before the local peak until 0.4 s after the peak were extracted as one segment, similar to [4]. The sample of extracted segments is shown in Figure 5b. Although some slight differences might occur, these segments can be assumed as an ideal BCG signal representation. Finally, the arithmetic mean from the segments was taken and utilized as the template of BCG signals for the following processes. The output of this process is shown in Figure 5c. The constructed template was then utilized for the subsequent processes.

### 3.3. Template Matching

In this step, the correlation coefficient between preprocessed BCG signals and the constructed BCG template were calculated. The computed coefficients were then scaled, such that the value ranged from −0.5 to 0.5. Since our interest was only in the positive correlation values, all the negative coefficients were treated as 0. The red curved line in Figure 5d is an example of the scaled correlation coefficients. From the output, the local maxima with minimum intervals of 0.7 s were again extracted and defined as heartbeats. The red circles on the red curved line in Figure 5d are the extracted heartbeats.

### 3.4. Evaluation

**Figure 6:**
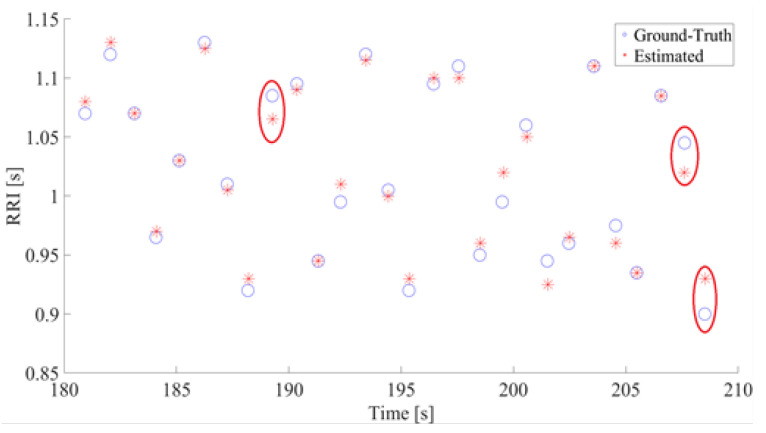
The Estimated RR and Truth RR (a sample of 30-s data). The Y and X axis show the RRI and time, respectively. In both figures, estimated RR is represented with asterisks and ground truth with circles. The instances where ground-truth and estimated RR differs by at least 0.02 s are circled in red.

All beat-to-beat intervals/HRV extracted from the defined peaks were calculated and utilized in validation. Signals used for validation were signals from sensors S1 and S2; in addition, the average signal of S1 and S2, and the average signal of all four sensors were also utilized for validation. The HRV calculated from these four signals was then divided for each 30-s segment because of the goal of the experiment. Although all of the training data were utilized in the template-matching approach, in this stage, several segments without corresponding PPG data were excluded. An example of the calculated HRV is shown in Figure 6. From these consecutive values, concordance analysis was performed using Bland–Altman plots and Lin’s Concordance Correlation. Additionally, three types of evaluation indices were computed: precision, root mean squared error (RMSE), and mean absolute error (MAE), were derived. The precision is the degree of correctness—how correct the detected HRVs were. It was calculated by the following simple equation.

(1) $$Precision=\frac{correct}{correct+incorrect}$$

All computed HRVs were classified into correct and incorrect categories using a 10% criterion. The HRVs calculated from the baseline (PPG) were defined as the correct answer. If the calculated HRV slipped more than 10% from the correct answer, that interval was defined as incorrect. Mean error means the average error between the correct answer and correctly estimated intervals. Since the error can be either negative or positive, common corrections to the mean error metrices are either to Equation (1) extract the square root of the squared error or Equation (2) apply absolute function/modulus to the error. The RMSE and MAE are computed by the following equations.

(2) $$RMSE=\sqrt{\frac{\sum_{i=1}^{n}{\left(y_{i}-x_{i}\right)}^{2}}{n}}$$

(3) $$MAE=\frac{\sum_{i=1}^{n}\left|y_{i}-x_{i}\right|}{n}$$

where y denotes the occurrence of true peak obtained from PPG and x is the predicted peak from this algorithm.

### 3.5. Sleep Stage Prediction

The most acceptable approach for determining sleep stage is visual inspection done by an expert [31]. Conventionally, the expert checks one epoch worth of PSG recordings of EEG, ECG, respiration rate, etc., and determines the sleep stage. An epoch is commonly set as 30 s or one minute. In our case, we tested multiple values of epochs, from 30 s to 270 s, with 30 s steps. The best result was obtained using an epoch of 30 s. Qualitative scoring of sleep stage is subjective, which might result in different results between different experts [32]. This leads to the development of automatic sleep stage classification. Unfortunately, conventional algorithms utilizing heart rate features did not have a high accuracy for five-stage sleep stage classification [28,29,30]. Some studies tried combine sleep stages to simplify the problem. Here, we proposed a mathematical model for automatic five-stage sleep stage classification using logistic regression, the parameters of which are optimized using the backpropagation algorithm.

To predict the sleep stage of the subject, multivariate logistic regression, expressed via the following equation, was performed.

(4) $$\sigma_{S}\left(\tau \right)=\frac{1}{1+e^{-(A+B\left[RR\left(\tau \right)\right]+C\left[RR_{std}\left(\tau \right)\right]+D\left[VLF\left(\tau \right)\right]+E\left[\frac{LF(\tau )}{HF(\tau )}\right])}}$$

where $\sigma_{S}\left(\tau \right)$ is the mathematical function to compute the probability of the patient is in sleep stage S during the time period of τ. Here, RR(τ), $RR_{std}\left(\tau \right)$, VLF(τ), LF(τ), and HF(τ) are the average R-R interval (RRI) (R-R interval; or HRV), standard deviation of RRI, the power of very low-frequency signals (0.0033–0.04 Hz), the power of low-frequency signals (0.04–0.15 Hz), and the power of high-frequency signals (0.15–0.4 Hz), respectively. A, B, C, D, and E are the parameters dependent on sleep stage S. The possible sleep stages are wakefulness (S = WK), rapid eye movement sleep (S = REM), light sleep (S = N1, N2) and deep sleep (S = N3). Here, τ can be set freely. The smallest theoretically possible τ is 1 s, although the recommended value is 30 s. The accuracy of the mathematical model is compared with the gold standard PSG. The five parameters utilized in this mathematical model are parameters commonly utilized in HRV research and we hypothesized that these parameters are appropriate for predicting sleep stages [33]. The frequency powers of the signal were obtained by Fast Fourier transform to a fixed-length window [34]. Before the feature extraction was performed, a 5-minute recording period was performed for initialization. For the first epoch, τ s of recording is additionally performed, and added to the initial recording. Then, VLF, LF, and HF features are extracted from the appended recording. The last 5 min of the recording are utilized for VLF extraction, the last 25 s for LF extraction, and the last 6 s for HF extraction. For example, for τ = 30 s and epoch = 1, we obtained a total of 5 minutes and 30 s worth of BCG data. The VLF is computed from the window from 30 s to 5 min 30 s. LF is computed from the window from 5 min 5 s to 5 min 30 s, and HF is computed from the window from 5 min 24 s to 5 min 30 s.

## 4. Results

Table 2 shows the results of the precision and data lengths used for the analysis and Table 3 shows the results of errors (RMSE and MAE). There are three notable points in our results: ‘sensors’, ‘postures’ and ‘subjects’.

An example of estimated HRV is shown in Figure 6. This figure shows the RR intervals between estimated R peaks and their occurrences in the time domain. As seen in the figure, the errors between our estimation and the ground truth are miniscule. In this example, the highest absolute errors were 25 ms for two pairs and 20 ms for one pair. The red circles indicate pairs of ground truths and estimated RR with absolute errors of 20 ms or more. From all comparisons between estimated RRs and ground truth RRs, the maximum absolute error was 30 ms and the minimum error was 0 ms.

The average precision from S1 is lower than that of S2 if the subject is lying on their back and if the subject is lying on their left side. This, however, is reversed when the subject is lying on their right side. To verify the statistical significance of the differences, *t*-tests were performed, and *p*-values were calculated. No significant differences (*p*-value ≥ 0.05) were found from the results. During the states where the subject lies on either side of their bed, however, the precisions are around 10% better when utilizing average S1 and S2. However, *t*-test results show no statistical significance for the improvements, except from the result of using S2 signals for subjects lying on their right side.

In all case of sleeping postures, the precision of utilizing the average of all sensors was inferior compared to utilizing individual signals. The *t*-test results also show significant differences between the average of all signals and individual sensor S1 for patients’ posture when lying on their back (*p* = 0.01) and patients’ posture when lying on their right side (*p* = 0.03). Significant differences were also found in the averages of all signals and individual signals from the S2 sensor for patients’ posture when lying on their back (*p* = 0.00).

The average precision of utilizing S1 and S2 for patients lying on their back tends to be higher than the other two postures. The results of a *t*-test with individual signals from sensor S1 show significant differences only between postures: lying on the back and lying on the left side. In the case of individual signals from S2, significant differences were found for patients’ posture when lying on their back, when lying on their right side, and when lying on their left side.

From the viewpoint of the signal used for the estimation of HRV, since no significant difference is found in the results of S1 and S2, it is considered that there is no difference in the SNR between the measurement signals of S1 and S2. The error rate of HRV, estimated from averaged signals of outputs from S1 and S2, is lower than the other cases and is the best among the other candidates. This result supports the results of our precision analysis. On the other hand, it also supports the results of the precision analysis in that the error rates increase when outputs of all sensors are averaged. From the standpoint of posture, a statistically significant increase in the error rate is observed for patients lying on their back and left side when using the average signals of S1 and S2, and for patients lying on their back and right side when using the average signals of all outputs.

From these results, it is revealed that the novel HRV estimation method in this paper, especially when using the average signal of S1 and S2, improves the accuracy and that it is possible to estimate HRV with a low error rate for any position or posture subjects adopt on a bed. Consequently, our system is considered to be effective for the daily monitoring of the cardiovascular system.

**Figure 7:**
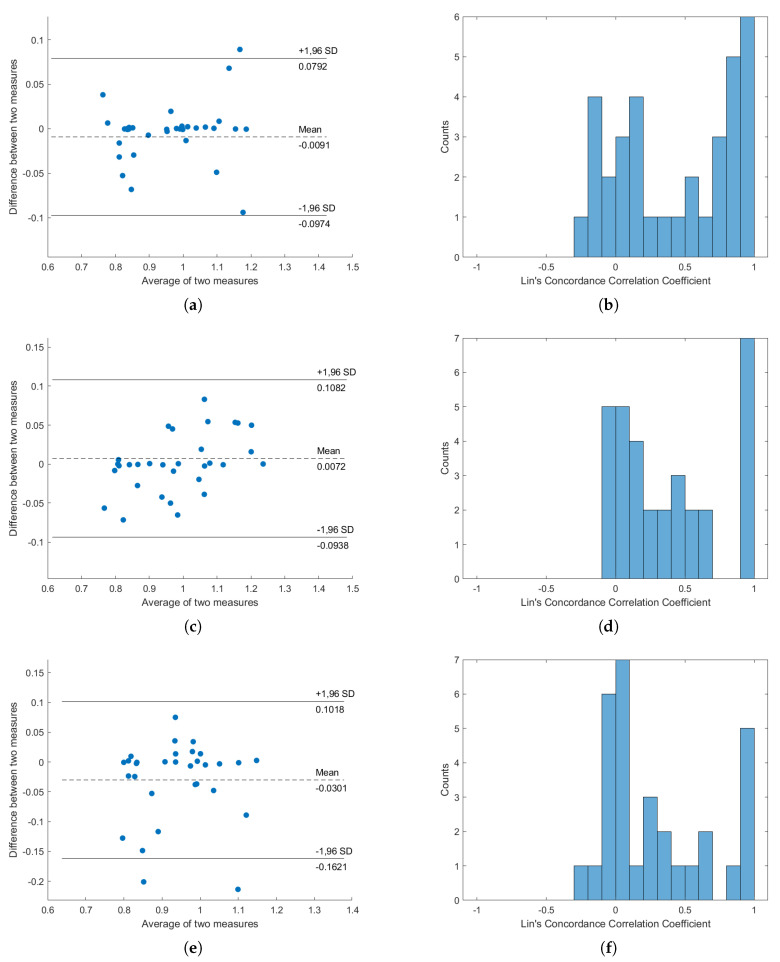
Concordance analysis result, from “Average signal of S1 and S2”. (**a**,**c**,**e**) are the Bland–Altman plots for subjects lying on their back, subjects lying on their left, and subjects lying on their right, respectively; (**b**,**d**,**f**) are the histograms of Lin’s Concordance Correlation Coefficients for subjects lying on their back, subjects lying on their left, and subjects lying on their right, respectively.

Bland–Altman analysis and Lin’s Concordance Correlation Coefficient from the average signals of S1 and S2 are shown in Figure 7, while the results from other sensors and postures are available in the Appendix A. From the results, it can be seen that, while the average signals of S1 and S2 have high concordance when the subjects lie on their back, the change in posture caused the concordance to drop. This effect is also observed in the histogram of Lin’s Correlation Coefficient. In all cases, the average signals of S1 and S2 seems to perform the best and the average signals of all signals seem to perform the worst.

The confusion matrix for sleep stage classification can be found on Table 4. This confusion matrix was a combined report from all of the folds from leave-one-subject-out evaluation; the sleep stages were checked per epoch and then combined by summation.

## 5. Discussion

No significant differences were found in the *t*-test of signals of S1 and S2. This implies that the SNR levels of signals obtained from sensors S1 and S2 are similar. The results of the precision analysis using solely S1 or S2 signals are worse compared to using average signals of S1 and S2, especially during the states where the subject lies on either side of their bed. During those cases, the precisions are around 10% better when utilizing average S1 and S2. However, *t*-test results show no statistical significance in the improvements, except for the result of using S2 signals for subjects lying on their right side. From these results, utilizing average signals of S1 and S2 seems to be the most effective method, presumably because arithmetically averaging the signals enhanced the SNR levels. However, further validation with more subjects is needed to confirm the effectiveness of utilizing the average signal.

From the results of the precision analysis, it can be concluded that the average signal of all sensors is not suitable for estimating HRV. The reason why the average of all signals is unsuitable might be because of the phase shift effect between signals from S1, S2, S3, and S4. The signals might have similar waveforms to each other, but the timings of the peaks-and-valleys occurrences differ. If the average of all sensors was to be utilized, this factor must be considered and the phase-shift effect must be examined thoroughly. If the shift is unvaried, the average of all signals might be suitable for HRV estimation, after applying a fixed phase shift to the signals.

Since the average precision of utilizing S1 and S2 for subjects lying on their back tends to be higher than the other two postures, it can be concluded that the subject’s posture relates to the accuracy of the HRV estimation. In particular, the cases where subjects were lying to on their right and left sides reduced the effectiveness of the estimation.

From the results of the concordance analysis, considerable concordance was found between HRVs extracted from PPG and HRVs extracted from BCG. The average signals from S1 and S2 show the most promising concordance and effectiveness, while the averages of all signals show the worst results. These results fit with the results of the precision analysis.

**Figure 8:**
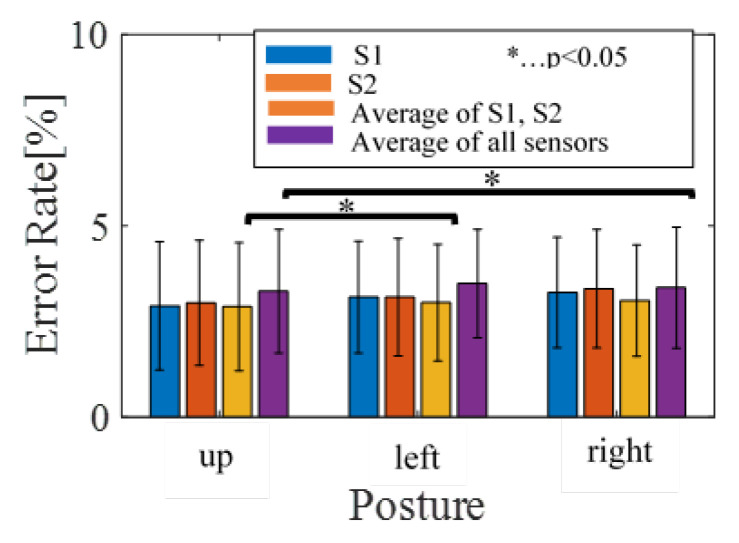
The average of error results.

The R-peak estimation algorithm is adequate, as shown in Figure 5 and Figure 6. The HRV estimation from those R-peaks also shows low quantitative errors of RMSE and MAE, and we deemed that the HRV estimation quality was enough for estimating the sleep stages. Overall, the HRVs were well obtained, even if individual differences were considered. The results from subject 3 have comparatively higher precision compared to other subjects, for all postures. The precision of subject 17, on the other hand, is inferior to other subjects for all postures. From these results, individual differences affecting BCG signals can be inferred. Figure 8 shows the average error results. In the figure, the average rates tended to be significantly lower than 5%.

**Figure 9:**
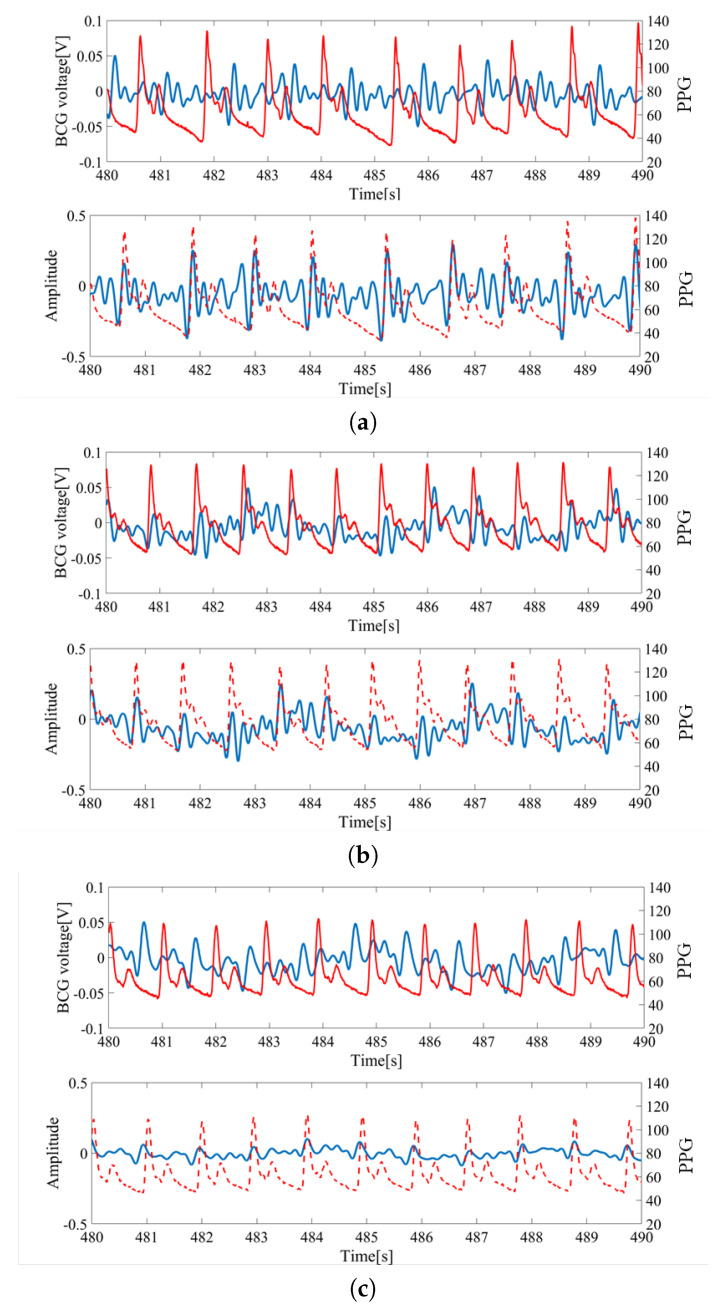
HRV extraction from the BCG; (**a**) sub1 (age: 30 s, height: 175 cm, weight: 59 kg, BMI 19.3). (**b**) sub5 (age: 20 s, height: 156 cm, weight: 52 kg, BMI 21.4). (**c**) sub14 (age: 30 s, height: 178 cm, weight: 90 kg, BMI 28.4). The blue line corresponds to raw data and preprocessed BCG data. The red line corresponds to finger pressure.

Although individual differences affected BCG data, our algorithm successfully acquired HRV by means of the analysis in Section 3. This can be confirmed from Figure 9a–c, which shows the HRV obtained from preprocessing steps. It is proven that satisfactory HRV can be obtained, even when considering individual differences.

From the interchangeability analysis results in Figure 7, it can be seen that the average difference (bias) between HRVs obtained from BCG and PPG are around 0, but high difference can be seen in some subjects, with few of them nearing the 1.96SD line. This shows that using the average BCG signals of S1 and S2 to compute HRV is inaccurate in general, but considerations of personal differences are still important and cannot be ignored. This is supported by the histogram of Concordance Correlation Coefficients (CCCs), as most of the correlation coefficients are either grouped as zero or one. This distribution suggests that the HRVs from PPG and HRVs from BCG are either highly correlated or highly uncorrelated, which might suggest that our subjects can be divided into two types of groups: one group which this proposed algorithm works very well against, and another group in which this proposed work is ineffective. Additionally, the averages of two measures are spread from around 0.8 to around 1.2, but were still within the boundary of normal HRV length. It must be noted, however, that, in this result, the minimum peak distance was set to be 0.7s, explaining the reason why the averages of two measures are always larger than 0.7.

From the results of Table 2, it is revealed that the accuracy of our novel HRV estimation method increases when using the average signals of S1 and S2, making low error HRV estimation with free bed positions or postures possible. Consequently, our novel system is effective for the daily monitoring of the cardiovascular system.

A logistic regression was performed to predict sleep stage based on average RRI, the standard deviation of RRI, power of very low-frequency signals, and the ratio of low-frequency powers vs. high-frequency powers. The second dataset of 100 recordings from 25 subjects was utilized to fit and validate the model with leave-one-subject-out cross validation. After the validation, a final regression model was created and the parameters are expressed in Table 5. The performance was measured by computing the accuracy of four-stage and five-stage sleep stages. In the case of the four-stage sleep stages, light sleep (N1 and N2) is classified into one category. The recognition accuracy was 89% for four-stage cases and 78% for five-stage cases, higher than conventional studies with 69% [27] and 67% accuracy [28,29,30]. This also shows that our HRV estimation algorithm is sufficient for sleep stage classification. However, it is important to mention that these reports are the reported effectiveness values in the papers. The database we utilized for this measurement is different from the conventional studies. Although our algorithm yields higher accuracy than the conventional algorithms, this does not necessarily mean that our algorithm is better, and there is a high chance that this high accuracy is simply caused by our dataset.

## 6. Conclusions

This paper consists of two parts: a proposal for a reliable method for estimating HRV from BCG, measured with bed sensors, and a mathematical model for sleep stage classification based on the obtained HRV. Our contributions are as follows:

1. In total, 86.9% of beat-to-beat intervals can be detected.
2. It is comparatively difficult to estimate HRV from BCG measured from subjects lying on their right and left sides.
3. There are individual differences in BCG.
4. It is better to use all sensors to detect heartbeats and estimate HRV upon taking into consideration the effects of the phase shift between signals.
5. Finally, a 78% accuracy is achieved for predicting sleep stage based on extracted beat-to-beat intervals.

In the field of BSN, there are many studies aimed at dealing with problems in BSN such as power consumption, latency, response time, the necessity of reliable and secure communication maintenance, and so on [21,22,23,35]. In these studies, a simple signal processing method in a sensor device or framework to reduce the amount of information to be transmitted to a server or mobile devices has been proposed. In contrast to these studies, since our system is a prototype of the bed sensor system, assuming wired power supply and use under a stable communication environment, this paper mainly focuses on only confirming that it is possible to estimate cardiac information reliably. In our future works, if necessary, we also aim to improve the communication efficiency, as in previous studies. Our system aims for real-time running implementation. To realize this goal, the development work for a real-time cardiac information monitoring application is currently underway.

Earlier versions of this paper were presented [36,37], with proposals of only measurement systems or HRV estimation methods. In those papers, experiments were conducted only on a small number of subjects and the recognition rate was not good. In the progress of our study, it was revealed that there are individual differences in the quality of measured BCG and, as a result of these individual differences, there are also individual differences in the estimation accuracy of HRV. In this paper, we extended the previous studies, proving that our method can cope with multiple people with robustness by increasing the number of people and improving the method, as shown in the results section. However, the results also show that the algorithm has a very low Concordance Correlation Coefficient for some cases. This shows the importance of personalization/fine-tuning the algorithm according to the active user. Another difference in this paper from previous papers was the proposal of a complete sleep monitoring system.

As the current study focuses on healthy subjects, one future consideration includes clinical experiments with non-healthy subjects. The improved method might be able to be utilized in clinical settings in hospitals, to detect heartbeat anomalies in inpatients. Currently, the plans to test our method in the heart rate acquisition of actual inpatients is scheduled, and the ethics review is in progress. The next step of this study will commence as soon as the ethics review board approves the proposal. Since the sensor can obtain heartbeat signals with no contact, it will be greatly beneficial in clinical practice.

A sleep stage prediction regression model was also proposed and, as a result, a mathematical model for predicting the sleep stage was obtained. The effectiveness of the mathematical model was tested for four-sleep stage classification and five-sleep stage classification and the observed accuracies were 89% and 78%, respectively. The next step will be to apply this algorithm to conventional databases and make a fair comparison to conventional algorithms.

## Figures and Tables

**Table 1:** Subject demographics for HRV extraction experiment and Sleep stage prediction experiment.

Parameter		HRV Extraction	Sleep Stage Prediction
N		36 subjects, 99 data	25 subjects, 100 data (4 trials per subject)
Sex		33 male, 3 female	14 male, 11 female
Age	20 s	10	22
	30 s	13	2
	40 s	13	1

**Table 2:** Results of precision (average ± standard deviation).

Position	S1	S2	Average of S1 and S2	Average of All Sensors
Back	0.81 ± 0.18	0.89 ± 0.15	0.89 ± 0.14	0.71 ± 0.20
Left	0.75 ± 0.16	0.75 ± 0.18	0.85 ± 0.18	0.65 ± 0.24
Right	0.79 ± 0.16	0.72 ± 0.15	0.87 ± 0.11	0.69 ± 0.20

**Table 3:** Results of error.

		RMSE [ms]	MAE [ms]
Back	S1	17.3	10.5
	S2	12.7	7.0
	Average of S1, S2	12.7	7.6
	Average of all sensors	20.3	13.8
Left	S1	21.5	12.8
	S2	18.1	10.8
	Average of S1, S2	14.8	8.5
	Average of all sensors	21.0	14.2
Right	S1	19.7	11.6
	S2	23.3	14.6
	Average of S1, S2	16.4	9.3
	Average of all sensors	23.1	14.9

**Table 4:** Confusion matrix.

	WAKE	REM	N1	N2	N3	Epoch Count
WAKE	153	41	0	12	15	221
REM	40	292	10	0	0	342
N1	13	13	102	14	11	153
N2	8	10	14	857	10	899
N3	0	0	7	22	213	242

**Table 5:** Parameters of sleep prediction.

	A	B	C	D	E
Wake	−2.84	−1.32	0.33	−0.20	−0.09
Rem	−1.80	−0.44	0.27	0.61	0.54
N1	−2.68	−0.15	0.24	0.15	0.25
N2	0.21	0.86	-0.32	−0.19	−0.11
N3	−2.71	0.23	−0.53	−0.64	−0.39

**Figure A1:**
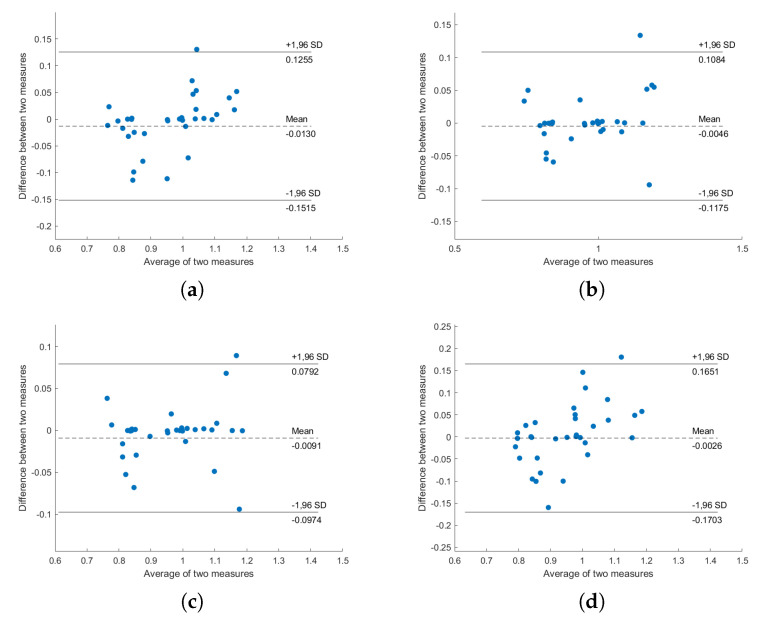
Bland–Altman plot of PPG-HRV and BCG-HRV, position “Back”: (**a**) S1; (**b**) S2; (**c**) S1 and S2; (**d**) all sensors.

**Figure A2:**
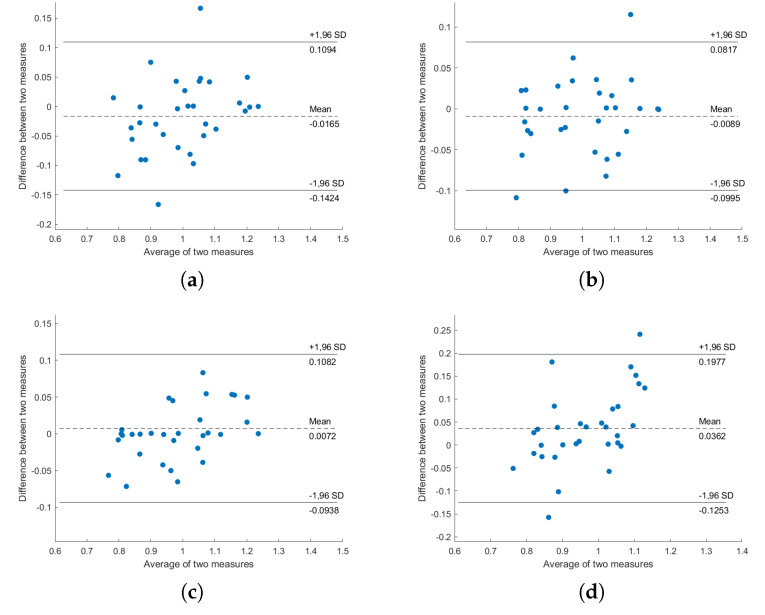
Bland–Altman plot of ECG-HRV and BCG-HRV, position “Left”: (**a**) S1; (**b**) S2; (**c**) S1 and S2; (**d**) all sensors.

**Figure A3:**
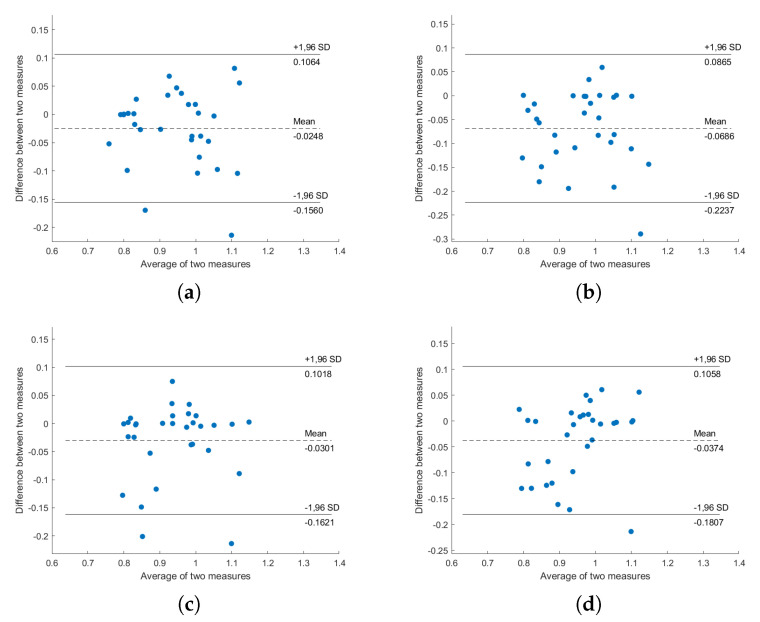
Bland–Altman plot of ECG-HRV and BCG-HRV, position “Right”: (**a**) S1; (**b**) S2; (**c**) S1 and S2; (**d**) all sensors.

**Figure A4:**
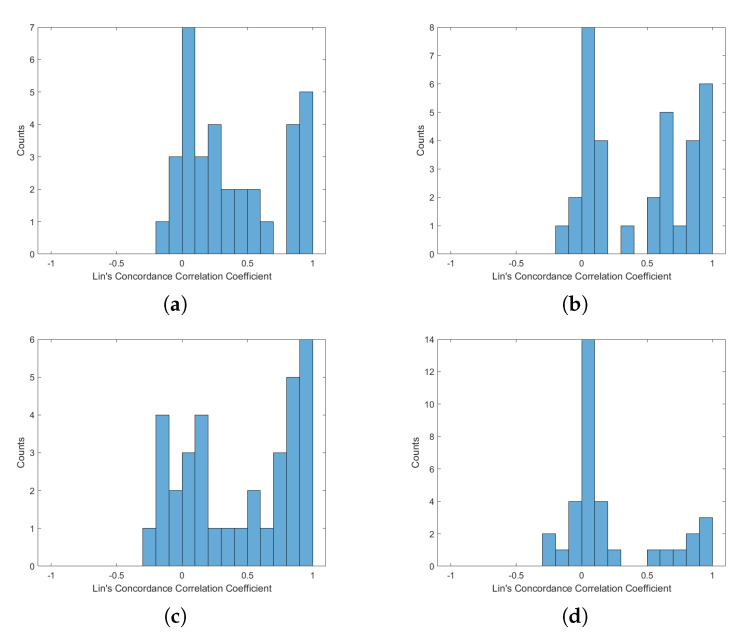
Histogram of Lin’s Concordance Correlation Coefficient from PPG-HRV and BCG-HRV, position “Up”: (**a**) S1; (**b**) S2; (**c**) S1 and S2; (**d**) all sensors.

**Figure A5:**
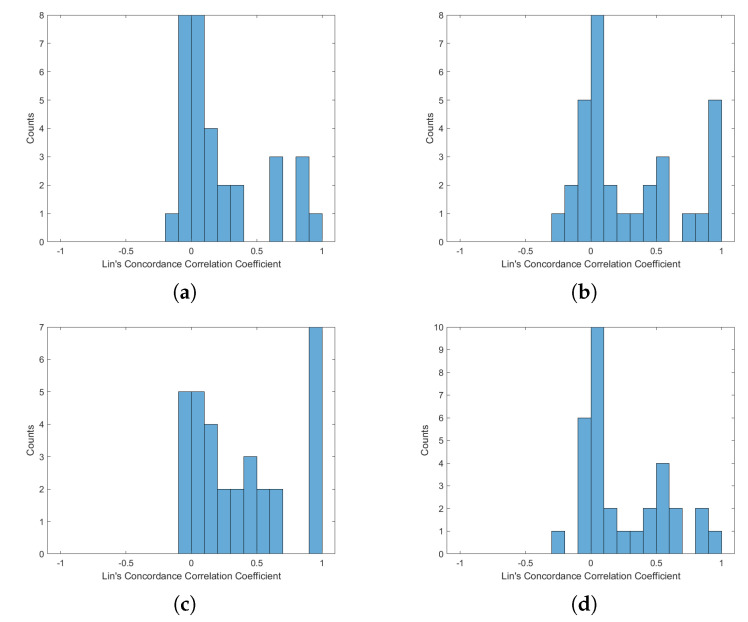
Histogram of Lin’s Concordance Correlation Coefficient from ECG-HRV and BCG-HRV, position “Left”: (**a**) S1; (**b**) S2; (**c**) S1 and S2; (**d**) all sensors.

**Figure A6:**
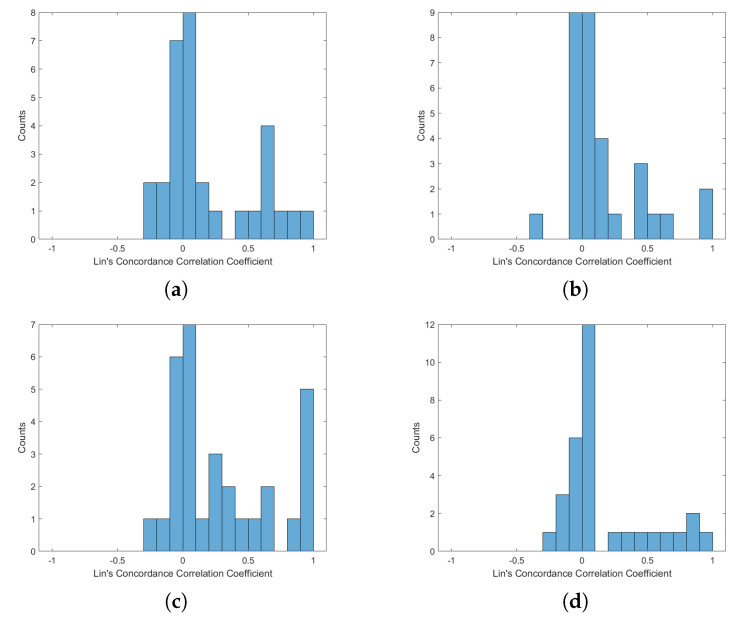
Histogram of Lin’s Concordance Correlation Coefficient from ECG-HRV and BCG-HRV, position “Right”: (**a**) S1; (**b**) S2; (**c**) S1 and S2; (**d**) all sensors.
